# Advancing Personalized Medicine Through the Application of Whole Exome Sequencing and Big Data Analytics

**DOI:** 10.3389/fgene.2019.00049

**Published:** 2019-02-12

**Authors:** Pawel Suwinski, ChuangKee Ong, Maurice H. T. Ling, Yang Ming Poh, Asif M. Khan, Hui San Ong

**Affiliations:** ^1^Malaysian Genomics Resource Centre Berhad, Kuala Lumpur, Malaysia; ^2^Centre for Bioinformatics, School of Data Sciences, Perdana University, Serdang, Malaysia; ^3^Centre of Genomics Research, Precision Medicine and Genomics, AstraZeneca UK Limited, London, United Kingdom; ^4^Graduate School of Medicine, Perdana University, Serdang, Malaysia

**Keywords:** big data, exome, personalized medicine, sequencing, precision, analytics

## Abstract

There is a growing attention toward personalized medicine. This is led by a fundamental shift from the ‘one size fits all’ paradigm for treatment of patients with conditions or predisposition to diseases, to one that embraces novel approaches, such as tailored target therapies, to achieve the best possible outcomes. Driven by these, several national and international genome projects have been initiated to reap the benefits of personalized medicine. Exome and targeted sequencing provide a balance between cost and benefit, in contrast to whole genome sequencing (WGS). Whole exome sequencing (WES) targets approximately 3% of the whole genome, which is the basis for protein-coding genes. Nonetheless, it has the characteristics of big data in large deployment. Herein, the application of WES and its relevance in advancing personalized medicine is reviewed. WES is mapped to Big Data “10 Vs” and the resulting challenges discussed. Application of existing biological databases and bioinformatics tools to address the bottleneck in data processing and analysis are presented, including the need for new generation big data analytics for the multi-omics challenges of personalized medicine. This includes the incorporation of artificial intelligence (AI) in the clinical utility landscape of genomic information, and future consideration to create a new frontier toward advancing the field of personalized medicine.

## Introduction

Advances in next generation sequencing (NGS) technologies have resulted in an unprecedented proliferation and deluge of genomic sequence data. Harnessing the information encoded in a person’s genome is far-reaching and has been instrumental in assessing the substantial portion of person-to-person variability in response to diagnosis, treatment, and prevention strategies (Seripa et al., 2010). This is done by comparing an individual’s genomic information to the DNA sequence of another “reference,” leading to a variability map of the population when done at a broader scale. The notion of individual variability dates back to Garrod, who in 1902 coined the term “chemical individuality” (Garrod, 1996). The definition has since become more precise; however, the “reference” still remains vague because of the heterogeneity that exists in the population genome (Huang et al., 2015; Lim et al., 2015; Spratt et al., 2016; Caswell-Jin et al., 2018). These genetic variations stand to impact significantly on the risk and survival outcome of a patient’s health (Dawood et al., 2008; Jemal et al., 2011; Eheman et al., 2012). This factor also points toward the potential challenges for advancing personalized medicine – in the hope of incorporating patient genetics within the management and treatment modalities toward better clinical outcomes.

The conventional approach of using candidate genes alone is not sufficient to explain the differences in disease risks that occur between ethnic groups, let alone individuals. The revolution of genotyping technologies has allowed focus on a specific region of the genome, thus enabling deeper coverage of the variants. This approach was successful in identifying prostate cancer risk loci (8q24 and 17q21) in men of African descent (Haiman et al., 2007, 2011; Yeager et al., 2007), which helped explicate the 50% increased risks of getting prostate cancer in these men (Amundadottir et al., 2006). In contrast to genotyping, the advent of the targeted sequencing approach has enabled the focus on specific regions of interest within the genome. This includes targeted amplicon sequencing and whole exome sequencing (WES). Going broader, the whole genome sequencing (WGS) approach provides the most comprehensive analyses of the entire genome; that is ~3 billion bases for a single “representative” haploid copy, in the case of a human. Notably, the complete set of protein-coding regions, the exome only constitutes ~3.09% (over 90 million nucleotides) of the latest release of the human reference genome, GRCh38 (Guo et al., 2017). Compared to WGS, targeted sequencing is a more cost-effective method and delivers a higher coverage, allowing for detection of rare variants. Coverage (breadth) for WES is referred to as capture of coding sequence targets (genes and their flanking regions) and in most cases include 22,000 genes. Coverage (depth) refers to the number of sequences for a locus based on independent reads. For clinical purposes, a target depth of 100× from Illumina machines is considered sufficient.

The lowest cost estimate for running a single WES test has fallen to £382 ($555) per exome, which is ~3.5 factor lower compared to the lowest cost estimate for WGS using HiSeq X (in Germany), £1,312 ($1,906) (Schwarze et al., 2018). This is in stark contrast to the cost per genome of ~$100 million, back in 2001 after the completion of the first Human Genome Project (National Human Genome Research Institute, 2016). The significant price reduction has taken the democratization of the sequencing to an entire new plateau.

Whole exome sequencing is attractive for clinical application mainly because it covers actionable areas of the genome to determine the variations in the exon regions and identify causal variants of a disease or disease-causing mutations (Gorski et al., 2016; LaHaye et al., 2016; Gambin et al., 2017; Gupta et al., 2017; Hixson et al., 2017; Mueller et al., 2018; Weigelt et al., 2018). There has been a tremendous boost in the generation of WES data at the population scale. The WES has proven its successful application in discovering of the gene associated with the Miller Syndrome, Mendelian phenotypes (Chong et al., 2015) and complex disorder (O’Roak et al., 2012; Jeste and Geschwind, 2014). Since 2011, WES has been routinely offered as a diagnostic tool in clinical genetics laboratories (Pierson et al., 2011; Yang et al., 2013). WES has since been incorporated into the 1000 Genome Project (Genomes Project et al., 2012), the NHLBI “Grand Opportunity” Exome Sequencing Project (GO-ESP) (Tennessen et al., 2012) and the efforts by the Exome Aggregation Consortium (ExAC) (Lek et al., 2016) to catalog population variants and to identify diseases associated with rare variants. These efforts bring us closer to the development of personalized medicine, by matching specific treatments to the genetic makeup of specific patients for maximum benefit. Recent breakthroughs heralding the new era for personalized medicine include approvals by the United States Food and Drug Administration (FDA) for monoclonal antibody pembrolizumab, targeting tumors expressing PD-L1 (Khoja et al., 2015) and olaparib, a poly(ADP-ribose) polymerase (PARP) inhibitor for ovarian cancer patients who carry mutations in BRCA1/2 genes (Rezende, 2014). More recently, the FDA approved larotrectinib (Vitrakvi), the first targeted therapeutic based on the tumor biomarker, instead of tumor origin in the body (Honey, 2018). The market size of personalized medicine is expected to reach USD 87.7 billion by the year 2023 (Newswire, 2016), while the digital genome market is expected to be worth over 45 billion by 2024 (Global Market Insights, 2017).

Herein, we review the application of WES genomic information in clinical practice. The review covers the big data characteristics of WES, discussing existing biological databases and bioinformatics tools to deal with the big data, including new generation artificial intelligence (AI) platforms. Concluding with the clinical utility landscape of genomic information, and future consideration to creating a new frontier toward advancing the field of personalized medicine.

## From Genetic Medicine to Genomic Medicine, Paving the Way for Personalized Medicine

The extent of genomic information utilization in medical practice is strongly linked to the advances in genomic technologies and sciences. The relatively small scope of clinical utility and the slow early uptake can be attributed to the lack of clinical evidence supporting the use of medical genomics in multifactorial diseases. Thus, the early focus on variants with high or near certain genotype–phenotype correlation probability (high penetrance) (Lobo, 2006). It soon became obvious that sequencing data alone is not sufficient to explain the genotype–phenotype correlation for multifactorial diseases, because they are characterized by a complex etiology, with variable genetic and environmental contributions. The genetic risk of developing multifactorial conditions is brought about by small and discrete alterations at the genomic/genic levels at multiple loci. Furthermore, these DNA changes exhibit low-to-medium penetrance power that is highly influenced by external factors related to the environment and lifestyle (Centre for Genetics Education, 2015; Abdullah Said et al., 2018). Knowing the sequence data was simply not enough to understand the etiological and pathogenic processes in complex diseases – the genomic data had a low predictive power and penetrance.

The sequencing of the human genome was more of a technological achievement rather than scientific. Knowing the exact position of all nucleic acids within the DNA molecule (99.9%) did not automatically mean we understand the functional implications of the sequence (Galas, 2001). To this end, several new initiatives were created to uncover the biological message behind the linear combination of the four nucleotides. One year before the full human genome was published, the HapMap project was launched to document the variations in the genome (Eichler et al., 2007). In 2005, the first GWAS was conducted to annotate medically documented genetic variants (Ikegawa, 2012). Soon, hundreds of studies were underway rapidly generating a clinical context for genomic data. The scope of GWAS was wide-ranging, but for most cases, focused on the risk factors and metabolic pathways related to multifactorial diseases. The GWAS contribution was crucial in uncovering strong polygenic-phenotype associations. Based on the GWAS discoveries, it was possible to identify essential metabolic pathways in many traits and medical conditions, paving the way for the first predictive and prognostic genetic test related to multifactorial diseases, and drug metabolism and response (pharmacogenetics).

In parallel, fast progress was made in researching for somatic variants from cancer cells. Neoplasms can be defined as acquired genetic diseases (~70% of all cancers), where the etiological genetic component is brought about by environmental factors (Malhotra et al., 2014). The cancerous transformation of cells is closely linked to genetic alterations in specific genes, e.g., proto-oncogenes, tumor suppressor genes, and DNA repair genes. It is possible to characterize the histological type of cancer cells based on the patterns of somatic mutations. This knowledge has been successfully explored to develop a range of cancer genetic tests:

• Predictive (e.g., testing BRCA1/2 genes for the genetic risk of developing Hereditary Breast and Ovarian Cancer) (McCartan and Chatterjee, 2018)
• Prognostic (metastatic potential and conventional treatment response) (Maman and Witz, 2018)
• Targeted treatments (small molecule therapeutics targeting specific gene mutations, e.g., imatinib for c-KIT gene mutation in Chronic Myeloid Leukemia and Gastrointestinal Stromal Tumors) (Druker et al., 2006; Grandori and Kemp, 2018)

Cancer genomics is a well-established field of medical practice and research. It is supported by strong clinical evidence and knowledge through many high-profile projects and initiatives such as:

• COSMIC (Catalogue of Somatic Mutations in Cancer) (Forbes et al., 2017)
• TCGA (The Cancer Genome Atlas) (Weinstein et al., 2013)
• ICGC (International Cancer Genome Consortium) (Zhang et al., 2011)

However, it took another 10 years, after the first GWAS was published, for research activities to elucidate the mechanisms underlying the genotype–phenotype association in multifactorial diseases. Since the environment is an essential modifier of the genetic effect, the inclusion of environmental and genetic factors as well as their combined effect on the downstream biological process in the assessment process is necessary to increase the predictive power of genetic alterations. In this approach, genes rather than single variants are assessed for their functional effect. It is a departure from the main GWAS assessment methodology, where the statistical association between the genetic variant and phenotype is measured without often accounting for the underlying biological process. It was a new concept that needed to be tested. In order to validate the clinical utility of functional genomic analysis, two sets of tools were required: (i) data from every layer of the molecular network involved in the translation of genetic effect to observed phenotype, and (ii) powerful computational tools capable of processing large volumes of data and making associations. It was to this end that numerous projects were launched, either to generate the data or to provide integrated bioinformatics tools for the clinical, functional analysis of the data.

## Big Data Characteristics of Whole Exome Sequencing

In 2025, genomics is expected to surpass the three biggest players in big data domains: Twitter, Astronomy, and YouTube (Stephens et al., 2015). Stephen’s team had mapped the key technologies that are needed to support big data genomics, in terms of data acquisition, storage, distribution and analysis. Data in genomics had also been mapped to the five Vs, characteristic of big data (He et al., 2017): volume, velocity, variety, veracity, and value. Below, we present the mapping of WES to not just the five, but the expanded 10 Vs of big data (Firican, 2017):

(i) **Volume** – **WES data size, which can vary by coverage and number of samples.** For the same sample at about 100× coverage, WES will generate ~5–6 GB of data. Although this is substantially lesser than ~90 GB for WGS (AllSeq, 2018) at the same coverage, the data size can grow substantially for a large number of subjects. Variant calling on exome sequence data in ExAC v0.3.1 from 60,706 individuals spanned 540 GB (Karczewski et al., 2017). Nowadays, many research studies involving tens of thousands of samples use WES for cost effectiveness, but it is clear that data generation is not the main issue, instead the bottleneck lies in data processing and analysis.
(ii) **Velocity** – **speed at which WES data per sample is generated and accumulated.** For example, a sequencing facility in 2013, equipped with 50 or so Illumina HiSeq 2000s and 2500s sequenced four exomes for every whole genome and had a capacity of some 2,000 exomes per week (Perkel, 2013). By 2018, the latest Illumina NovaSeq 6000 System is able to sequence a human genome (30×, >120 GB) at a pace of every 55 min, and an exome (100×, ~8 GB) every ~5 min (Illumina, 2018). This empowers users to high-throughput sequence up to 48 human genomes or close to 500 exomes per run in less than 45 h.
(iii) **Variety** – **the different attributes of WES data.** One aspect of this can be in terms of the five Ws and one H: what (WES), who (gender/age/ethnicity), why (diseased versus healthy), where (organ/tissue/cell), when (day/month/year), and how (accuracy/coverage)? For example, a 100× (how?) WES dataset (what?) can be generated from a centenarian (who?) with tumor (why?) in the neck and bladder (where?) that is in the late stage (when?).
(iv) **Veracity – confidence or trustability in WES data.** Various sources of errors and confounding factors can affect the confidence or trustability of the sequencing data. For example, because of mismapped shortreads, mosaicism, and sequencing errors, variant callers can end up predicting close to sevenfold more than the ~3 million variants in an individual human genotype (Robasky et al., 2014). It is challenging to differentiate small mutations from random errors generated during sequencing (Hofmann et al., 2017). Additionally, a major shortcoming of WES is the uneven coverage of sequence reads over the exome targets, contributing to many low coverage regions, which affect the downstream analysis, and thus, hinder accurate variant calling (Wang Q. et al., 2017). For example, some regions are still poorly captured (coverage as low as 10×) in a sample with a high average read depth (>75×), which can cause potentially out-turn in missed variant calls (Hoischen et al., 2014).
(v) **Variability** – **inconsistencies and multitude of dimensions in WES data.** Inconsistencies can include anomalies and outliers, which can be picked up using analytical methods; it can also include inconsistent speed at which data is loaded into the repository. A patient could have a totally or partially rearranged genome as seen in those with autism in one extreme of anomaly (Tabet et al., 2015). Multiple data dimensions can result from disparate data types and sources.
(vi) **Validity** – **WES data accuracy and readiness for analysis.** In 2017, the accuracy of various variants calling pipelines was investigated for exome sequencing by the PrecisionFDA Hidden Treasures – Warm Up challenge, a contest run by the FDA to promote more accurate genetic screening (PrecisionFDA, 2017). Edico DRAGEN received the highest overall score and Saphetor was the second. Besides a choice in computational pipeline, sequencing artifact can also affect the search for reliable results in the exome sequencing data, particularly in identifying the properties that distinguish false positive variants from true variants. To overcome this, a trio design strategy (father, mother and child) had been used to filter out (removing sequencing artifacts) and retain true mutations (Patel et al., 2014). As for readiness from raw data to analysis, for example, DeepVariant, using Google Cloud, can take ~70 min (time estimate does not include mapping) for a whole genome at 30× coverage, and ~25 min for an exome (DeepVariant, 2016).
(vii) **Vulnerability** – **WES data security and data breach.** Human genomic data has the potential to reveal sensitive information and is potentially re-identifiable, as such privacy and security are often at risk. Several studies have reported vulnerability of the human genomic data, which enables re-identification of patients from an ‘anonymous database’ (Homer et al., 2008; Gymrek et al., 2013; Harmanci and Gerstein, 2016). Shringarpure and Bustamante (2015) demonstrated that an individual can be re-identified by repeatedly querying the genome data sets via an open-access Beacon for alleles associated with an individual’s genome. Moreover, there are also concerns surrounding the policy and practice of returning genome sequences back to research participants (Wright et al., 2017), whereby substantial resources are required to ensure the safety return of that whole data to individual participants (Kaye et al., 2014).
(viii) **Volatility** – **how long before the WES data is considered obsolete or irrelevant.** Currently, the driving factor behind WES is the favorable cost, when compared to WGS. WGS is more powerful than WES for the detection of potential disease-causing mutation within WES regions, especially in those regions due to single nucleotide variants (SNVs) (Belkadi et al., 2015). Additionally, WGS is also more comprehensive than WES, and thus more useful when the disease causing variant is not in the exome, as in the case of limb malformation due to mutation in the limb enhancer of sonic hedgehog gene (SHH) (Visel et al., 2009). Thus, in the future, when the cost for WGS reduces to the point of being equivalent or lower than the current cost of WES, then the relevance of WES data becomes questionable. Thus, one may consider that the attractiveness of WES for clinical use is of a limited shelf-life, subject to WGS becoming affordable to the masses. It is estimated that by 2020 or later, the cost for WGS may be as low as USD 100 (Herper, 2017).
(ix) **Visualization** – **how challenging it is to visualize WES data**. Visualization of sequence data is an important tool for researchers and clinicians, especially those without extensive IT skills. Exome data is currently visualized through various popular browsers (Table 1) that provide a gene- and transcript-centric display of variation (Karczewski et al., 2017), with extensive functionality for comparative analysis, as well aggregation of available knowledge. However, plotting graphical representation of NGS data in real-time comes with a cost: higher computational requirements (computing power, memory, and storage) and faster Internet. Additionally, many of the genome Internet viewer use older annotation databases than those installed locally, which might be a significant restriction. For example, viewers only accepting sequences aligned to GRCh37/hg19 assembly (current version GRCh38), support dbSNP version 141 (current version is 151), and Ensembl VEP 85 (current version 94). Increasing complexity of viewing data adds additional needs for storage, as in the example of the 3D Genome Browser requiring at least 10 GB for compressed data (1T for uncompressed). Newer genome viewers utilizing cloud computing technology are gaining popularity as they provide good resource optimization, satisfactory performance and affordability for those requiring commercial license (e.g., DNAnexus).

**Table 1:** List of biological databases and bioinformatics tools relevant for data-warehousing, alignment, processing or analysis of sequence reads.

Category	Bioinformatics tools	Reference
Read alignment	BWA	Li and Durbin, 2009
	Bowtie	Langmead, 2010
Annotation	Annovar (Qiagen)	Qiagen, 2018a
	Variant Effect Predictor (Ensembl)	McLaren et al., 2016
	SNPsift and SNPeffect	Cingolani et al., 2012
	Variant Annotation Integrator (UCSC)	Hinrichs et al., 2016
	NCBI Variant Annotation	Church et al., 2013
	Sift4G	Vaser et al., 2016
	WGS annotator (runnable on the Amazon Compute Cloud)	Liu et al., 2016a
Visualization	NCBI Variant Viewer	National Center for Biotechnology Information, 2018
	UCSC Genome Browser	Kent et al., 2002
	ENSEMBL Genome Browser	Stalker et al., 2004
	ExAC browser	Karczewski et al., 2017
	Integrative Genomics Viewer (IGV)	Thorvaldsdottir et al., 2013
	Personal Genome Browser (PGB)	Juan et al., 2014
	3D Genome Browser	Wang et al., 2018b
Data-warehousing	ClinVar (clinical significance)	Landrum et al., 2014
	dbSNP (NCBI main variant annotation database)	Sherry et al., 2001
	dbNSFP (variants damage prediction using many *in silico* algorithms)	Liu et al., 2011
	COSMIC (Catalogue of Somatic Mutations in Cancer)	Forbes et al., 2017
	GWAS Catalog	Welter et al., 2014
	GWAS Central	Beck et al., 2014
	Cancer Atlas	Liu et al., 2018
	RefSeq	Pruitt et al., 2005
	PANTHER	Thomas et al., 2003
	TCGA (The Cancer Genome Atlas)	Weinstein et al., 2013
	ICGC (International Cancer Genome Consortium	Zhang et al., 2011
Analytics	Genome Analysis Toolkit (GATK)	DePristo et al., 2011
	MuTect	Cibulskis et al., 2013
	OTG-snpcaller	Zhu et al., 2014
	ASEQ	Romanel et al., 2015
	Halvade-RNA	Decap et al., 2017
	GT-WGS	Wang et al., 2018a
	EXCAVATOR2	D’Aurizio et al., 2016
	KaryoScan	Maxwell et al., 2017
AI-based analytics	Exomiser	Smedley et al., 2015
	DeepVariant	Knight, 2017
	Deep Genomics	Knight, 2017
	Qiagen (Ingenuity Variant Analysis and Ingenuity Pathway Analysis)	QIAGEN, 2018b
	Golden Helix (VarSeq, VSCkinical)	Golden Helix, 2017
	Advaita (iVariant/iPatway/iBio Guides)	ADVAITA, 2018
	Lifemap Sciences	TGex^TM^, 2018

(x) **Value** – **usefulness of WES data.** Genomic data has clearly established its fundamental value, while exome data as a focus on the coding sequences does have its contribution in improving health outcomes. For example, WES provides value to the medical system through better ability to give patient-directed care, to anticipate future medical needs and avoid unnecessary interventions. As a diagnosis to a family, it diminishes the need for other testing; and allows new gene discovery and re-analysis of old data with new information (Mayo Clinic, 2017).

**FIGURE 1:**
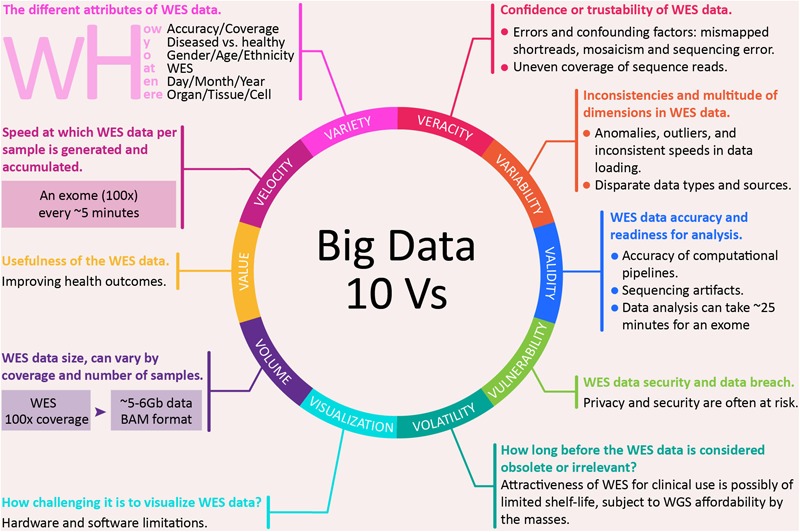
The 10 Vs big data characteristics of whole exome sequencing.

The 10 Vs, characteristic of big data are applicable to WES (Figure 1), and thus, they naturally extend to WGS. The value each sequencing approach brings would be useful at different levels. The limitation of WES, however, relative to WGS is the focus on the coding sequences. With the expected cost reduction of WGS, it remains to be seen if WES remains useful for discovery and statistical analysis. Nonetheless, targeted sequencing, both WES and amplicon, are expected to remain relevant, similar to genotyping, as a way to concentrate the research resources, akin to “less is more.”

## New Generation of Big Data Analytics

### NGS Technological Platforms and Approaches

The completion of the human genome project marked the start of an era of significant growth in genome sequencing technologies, termed as “Next Generation Sequencing.” This resulted in various NGS techniques, besides WGS and WES, such as RNA-seq, Chip-seq, and Bisulfite-seq and the accompanying development of tools for data analysis (Table 2).

**Table 2:** Comparison of various NGS technique and primary analysis tools.

NGS techniques	Study aim(s)	Data size per sample	Tool(s) used	Reference
WGS	*De novo* assembly	~90 GB	Velvet, SOAPdenovo	Zerbino and Birney, 2008; Luo et al., 2012
WES	Protein-coding variant identification	~5–6 GB	Edico DRAGEN, GATK, Samtools	Li et al., 2009; McKenna et al., 2010; Edico Genome, 2018
RNA-seq	Gene expression, novel isoform discovery	~3–4 GB	DESeq, Cufflinks	Anders and Huber, 2010; Trapnell et al., 2012
ChIP-seq	Protein–DNA interaction study, i.e., identification of histone marks and transcription factor binding sites	~1–2 GB	QuEST, MACS	Valouev et al., 2008; Liu, 2014
Bisulfite-seq	DNA methylation sites identification	~1–2 GB	BS Seeker	Chen et al., 2010

There are currently two major approaches in NGS technology, whether performing WES or WGS. Short read sequencing approach, such as by use of Illumina HiSeq X, provides a reduced cost and higher accuracy data, which are geared toward population level studies and clinical variant discovery, whilst, long read approaches, such as by use of PacBio’s single molecule real-time (SMRT) sequencing machines, are designed more for *de novo* genome assembly applications or isoforms discovery (Goodwin et al., 2016). Short read massive parallel sequencing has emerged as a standard tool for clinical use (Ardui et al., 2018). However, there are inherent limitations, such as GC bias, difficulties mapping to repetitive elements, trouble discriminating paralogous sequences, and difficulties in phasing alleles. These obstacles can be addressed by long read single molecule sequencers. Additionally, they offer higher consensus accuracies and detection of epigenetic modifications. Nonetheless, their utility in the clinical setting has been limited because of low throughput and high cost.

The WES data can be obtained using different technological platforms. First generation sequencing, e.g., Sanger sequencing, is based on chain termination and electrophoretic separation for the detection of newly incorporated nucleotide. It is a slow and costly process, but a highly accurate method. It is routinely used for confirmation of genomic alteration discovered by other methods (Sanger et al., 1977). To speed up the sequencing process, new technology was developed that uses chemical reaction and optical detection in a massive parallel process. These technologies are often called the NGS or second generation sequencing and include proprietary methods, such as sequencing by synthesis (SOLEXA/Illumina), sequencing by ligation (SOLiD/Life Technologies), pyrosequencing (454/Roche), and semiconductor sequencing (Ion Torrent) (Buermans and den Dunnen, 2014; Kchouk et al., 2017). Each of them has specific application based on their advantages and weak points. Very often the use of these technologies is determined by the length of the reads length, the accuracy of base calling, and the cost per base-pair. Third generation of sequencing technology is characterized by departure from amplification via sequencing of just one DNA molecule (or one cell DNA) using physical properties of DNA. Oxford Nanopore is one of the industry leading companies that commercialized the technology, which uses electrical impedance to detect the nucleotide passing through a membrane. Some sources distinguish 4th generation of sequencing technology for real-time single molecule sequencing (SMaRT). Although they accuracy is still below the second generation sequencing machines, they are the perfect tools for point-of-care (Laver et al., 2015; Lee et al., 2016).

When performing WES, a key consideration factor is the selection of the exome capture kit, more than the choice of platform. Various commercials kits are available, such as Agilent SureSelect XT, Agilent SureSelect QXT, NimbleGen SeqCap EZ and Illumina Nextera Rapid Capture Exome. They use biotinylated DNA or RNA baits, which are hybridized to genomic fragment libraries. Yet they differ in target region selection, bait length, bait density, molecule used for capture and genomic fragmentation method. If the aim is to detect SNVs and indels in untranslated regions (UTRs), then NimbleGen platform stands-out, while both Agilent XT and Illumina perform similarly for SNV and indel detection in coding regions (Shigemizu et al., 2015).

### NGS Data Analysis

Medical conditions that are genetically determined or have a strong genetic component arise from a variety of DNA alterations. These molecular events include SNV [referred to as single nucleotide polymorphism (SNP)] if they occur to some appreciable degree (>1%) in a population) and structural DNA changes, such as copy number variation (CNV), short insertions and deletions (indels), repetitions, large insertions and deletions, translocations (can result in fusion genes), inversions, aneuploidy, and ploidy (Ye et al., 2016). WES is primarily used for the detection of SNV/SNPs and indels within the coding regions of a genome.

Massive parallel sequencing of short reads through NGS generate big data, which has to be aligned (mapped to a reference genome or generate *de novo* genome sequence) for analysis. When a reference genome is available, the first step in data analysis is mapping the reads onto the reference genome (Shang et al., 2014). The intention is to “stack” each reads on the reference genome “floorplan.” If the template molecules are mRNA (thus, known as RNA-seq), the “height” of each stack corresponds to the abundance of mRNA for the specific genomic locus (Conesa et al., 2016; Zhao et al., 2018), at the resolution of each nucleotide. In the case where the template molecules are DNA (thus, known as DNA-seq), the “height” of the stack corresponds to the multiple of copy number and number of haploids. In this case, SNPs is rendered as mismatches on the stack (Kumar et al., 2012). In an event where a reference genome is not available, *de novo* assembly or genome annotation can be used. *De novo* assembly is based on the premise that each read may be overlapping and can be used to generate a contig assembly (Cho et al., 2015; Deng et al., 2015; de Sá et al., 2018), much like an assembly from shotgun sequencing (Staden, 1979; Hung et al., 2013). Once a contig is rendered, it can be used as a proxy to a reference genome. Genome annotation (Nagasaki et al., 2013; Menon et al., 2016), on the other hand, is direct analysis of the reads by two steps. In the first step, each read is annotated using tools such as BLAST (Altschul et al., 1990), functional annotations using tools such as InterProScan (Jones et al., 2014), or pathways by sequence similarities to known enzymes. This is sometimes known as read annotation. This is followed by the second step during which reads are mapped onto a scaffold; such as, a genome or a pathway map. When mapped by BLAST to another genome; for example, BLAST of *Bacillus subtilis* NGS data to *Escherichia coli* genome; then *E. coli* genome can be used as a reference genome. As such, there are commonalities between all these methods (mapping to reference genome, *de novo* sequencing, and genome annotation) of data analysis as the end result requires the mapping of reads onto some form of scaffolding substrate. When NGS data is functionally annotated to known proteins or pathways, the set of proteins or pathways will be used as a reference and transcript abundance or SNP calls can be made.

The Broad Institute had developed a set of tools, the Genome Analysis Toolkit or GATK (DePristo et al., 2011), for analysis of reads with the ability to combine various tools within GATK into a workflow for better documentation and reproducibility. GATK can be accessed at https://software.broadinstitute.org/gatk and various example workflows are also publicly available at https://software.broadinstitute.org/gatk. As more tools are added to do GATK, the possibility of workflows is virtually endless. For example, do Valle et al. (2016) had combined GATK and MuTect (Cibulskis et al., 2013), another tool by the Broad Institute and had been included into GATK, for more accurate SNP calls. Hence, it is foreseeable that combinations of existing tools may yield better results than individual tools, which also demonstrates the advantage of workflows. A volume of recent studies (Ahn et al., 2016; Engelhardt et al., 2017; Kim et al., 2017; Coudray et al., 2018; Han et al., 2018) had used GATK for mutation/SNP analysis using WES data. For example, Artomov et al. (2017) performed WES on more than 10,000 patients and analyzed the data using GATK to identify rare variants in hereditary melanoma. From this study, a mutational landscape of cutaneous and ocular melanoma, and implicated Early B Cell Factor 3 (EBF3) as a potential cutaneous melanoma pre-deposition gene. Table 1 provides a list of biological databases and bioinformatics tools relevant for data-warehousing, alignment, processing or analysis of sequence reads.

Since GATK and MuTect, several other tools had been published, including a number that utilize GATK. For example, OTG-snpcaller (Zhu et al., 2014) combined Ion Torrent’s Mapping Alignment Program (TMAP) and GATK for SNP calls. This had been used in WES analyses, leading to the identification of a missense mutation in sodium voltage-gated channel alpha subunit 8 (SCN8A) in a clinical presentation of early infantile epileptic encephalopathy type 13 (Malcolmson et al., 2016). ASEQ (Romanel et al., 2015) is designed to perform gene-level allele-specific expression analysis from genomic and transcriptomic NGS data to identify allele specific features, and had been used to analyze chemotherapy-resistant urothelial carcinoma for insight that can be used to develop new treatment modalities (Faltas et al., 2016). Halvade-RNA (Decap et al., 2017) re-implements GATK workflow to take advantage of parallel processing to reduce processing time and achieve 93.8% overlaps in variant identification.

Besides SNP calls, tools for detecting structural variations are also developed. For example, CNNdel (Wang J. et al., 2017) uses convolutional neural network on the output from various feature analysis tools to identify structural variations. GT-WGS (Wang et al., 2018a) takes advantage of Amazon Web Services to process NGS data and achieves 99.9% consistency with GATK best practice in SNP and indel calls. CNVs and larger structural changes still can be identified as long as they are limited to exonic regions. This is possible through the application of bioinformatic algorithms capable of accurately measuring read’s depth and allelic imbalances in the aligned sequence (BAM file). EXCAVATOR2 and KaryoScan are examples of such methods with the former being able to detect CNVs and the latter large chromosomal aberrations and changes to chromosome numbers (D’Aurizio et al., 2016; Maxwell et al., 2017). WES is not recommended to be used for translocations and repetitions (e.g., tandem repeats), because of their tendency of having break-points or extending beyond genic space (Belkadi et al., 2015).

### New Generation Analytics for Multi-Omics Big Data

Although data generation is not an issue with the advent of NGS and there are bioinformatics tools and databases to handle the resulting big data, the upcoming long read, single DNA molecule sequencing, such as the Oxford Nanopore, can offset the volume of data generation from the second generation NGS. However, while the sequencing data can be decreased, the omics data needed for personalized medicine presents higher complexity and is more voluminous than second-generation sequencing data, and would require continuous evolution or new generation of bioinformatics tools and data-warehousing approaches. For example, in April 2016, AstraZeneca announced an integrative genomics initiative to transform drug discovery and development by delivering novel insights into the biology of diseases, identifying new drug targets, supporting patients’ selection for clinical trials and matching patients to the therapies most likely to benefit them, a.k.a personalized medicine (Gameiro, 2016). The initiative included collaborations with Human Longevity, The Wellcome Trust Sanger Institute, United Kingdom, and The Institute for Molecular Medicine, Finland. In order to deliver the bold initiative, AstraZeneca established an in-house Centre for Genomics Research, which will sequence and analyze up to two million genome sequences (WGS and WES), including 500,000 samples from their clinical trials by 2026. Working in collaboration with DNAnexus (Business Wire, 2017), the use of a secure cloud-based translational informatics platform was adopted (Business Wire, 2017) to allow for warehousing and analyses of unprecedented massive volume of raw sequencing data rapidly and economically. This was aimed at enabling the processing of samples from thousands of patients per week and the sharing of data easily and safely with collaborators around the world. The platform also provides a secure environment where genetic data can be combined with de-identified clinical data, paving the way for novel scientific insights.

## The Clinical Utility Landscape of Genomic Information

### Pharmacogenetics

Personalized medicine, as the tailoring of clinical interventions, is mostly pharmacological, based on a person’s ability to respond favorably; for pharmacological agents this entails metabolic capability to process them. The CYP450 family of enzymes are responsible for phase one of xenobiotics metabolism, and their activity can be altered by genetic variants located in their respective genes. Identifying such genetic variants can help in predicting drugs’ pharmacokinetics and pharmacodynamics, which can then assist clinicians in selection of interventions that will achieve desirable therapeutic effect without toxicity (Evans and Relling, 2004; Feero et al., 2008; Whirl-Carrillo et al., 2012; Carr et al., 2014). For drugs with a narrow therapeutic range, such as blood-thinning agents, a small functional activity change can result in either a too low or a too high physiological effect that can lead to health complications. Adverse drug reactions (ADRs) are reported to be one of the major causes of morbidity and mortality that can easily be avoided. In the United States, 3% of registered drugs carry FDA recommendation for genetic tests (FDA, 2018).

### Cancer Therapeutics

Besides predicting the response to common drugs, genetic information is also used in matching targeted cancer therapeutics (Johannessen and Boehm, 2017). While pharmacogenetics for common drugs detects germline variants, cancer pharmacogenetics is for selecting small molecule inhibitors and analyzing somatic variants from tumor cells. As cancer is predominantly a genetic disease, tumor DNA analysis is routinely deployed for molecular characterization of the cancer cells, as well as treatment prognostics and monitoring. Obtaining tumor samples for genetic analysis can be a challenge if the growth is small or inaccessible. In recent years, liquid biopsy has been successfully applied to obtain a tumor circulating free DNA. It is now possible to use liquid biopsy for early cancer detection, prognostics, and treatment selection and monitoring. Unfortunately, the cost of cancer genetic tests and targeted treatments are still very high, making them inaccessible in less developed countries.

### Reproductive Health

Reproductive health is another area that has benefited from WGS and WES. Shallow WGS (3X) is performed for preimplantation assessment of embryos. It is also used for gender selection. Non-Invasive Prenatal Test (NIPT) is a combination of liquid biopsy and WGS for the detection of trisomies or other large chromosomal rearrangements in the fetus cells. It is possible to replace WGS with a higher coverage WES for both tests, which could make the tests more affordable (Pray, 2008).

### Multifactorial Diseases

The clinical utility of genomic information for multifactorial diseases still lacks enough predictive power and strong scientific evidence. However, the advances in bioinformatics technologies, allowing multi-omics analysis, is showing promising results. There are already reports about polygenic risk score for complex medical conditions attaining similar predictive power as genetic risk assessment for monogenic diseases (Khera et al., 2018).

## The Rise of Artificial Intelligence

### AI-Driven Genomics

High costs and limitations in terms of technologies have remained the main barriers for the greater omics-based implementation of personalized medicine. AI-driven machines, are being deployed to cut costs, especially in overcoming the enormous volume of collected patient data. For instance, Congenica’s Sapientia uses the Exomiser tool to accelerate the annotation and prioritization of variants from whole-exome sequencing in the diagnosis of rare diseases (Smedley et al., 2015). Sapientia empowers clinical decision-making by organizing the data into an easily comprehensible fashion, which helps to cut diagnosis times down from 5 years to 5 days (Congenica, 2018). AI-driven machines are even predicted to perform better than humans, from driving a truck (as autonomous vehicles) by 2027, writing a bestselling book by 2049, to performing a surgery by 2053 (Grace et al., 2018).

Meanwhile, tech giants, such as Google and its competitors are furiously adding machine-learning features to their cloud platforms in an effort to attract people to tap into the latest AI techniques (Knight, 2017). For instance, Deep Genomics uses deep learning to tease out genetic causes of diseases and potential drug therapies, and Wuxi’s Nextcode, which invested heavily in machine learning methods, are among the companies behind such efforts.

The Google Brain team, a group that focuses on developing an AI application and Verily, another Alphabet subsidiary that focuses on life sciences, released a tool known as DeepVariant that uses the latest AI techniques to construct a more accurate picture of a person’s genome from their sequencing data (Knight, 2017). It automatically identifies insertion, deletion and single-base-pair mutation in sequencing data. Millions of high-throughput reads and genomes from the Genome in a Bottle (BIAB) project, No Author (2015), were collected to feed the data to the deep-learning system and the parameters of the model was painstakingly tweaked until it learned to interpret the sequences data with a high level of accuracy (Knight, 2017). In 2016, DeepVariant won the first place in the PrecisionFDA Truth Challenge, in the best SNP performance category, and thus highly accurate. DeepVariant is also extensively fast, robust, cost efficient, flexible, easy to use, and where you need it by using Google Cloud Platform (DeepVariant, 2016).

### Omics Analytics Powered by AI Technologies

AI can improve statistical computation, but it needs more data to do the guess-work (Lopes et al., 2012; Topol, 2014; Carter and He, 2016; Camacho et al., 2018). Although the size of NGS data is significantly dropping, thanks to the introduction of single-molecule sequencing (Oxford Nanopore) (Rabbani et al., 2014; Halvaei et al., 2018), the downstream AI analysis requires exponential volumes of longitudinal data for making the genotype–phenotype connection as accurate as possible. While the quest for more robust causal algorithms is underway, a number of bioinformatics tools have been developed aiming to link sequence variants with biological metadata and phenotype. These new generation tools provide *in silico* assessment of omics data, derived from WGS or WES, and analytical capacity (often deploying AI) for variants prioritization/phenotype scoring (Shihab et al., 2013; Liu et al., 2016b). This approach has already proven to generate sufficient predictive power that can be compared to the prediction of Mendelian diseases (Khera et al., 2018). The next step entails the translation of scientific findings into easily understood medical standards, similarly to how pathology test results are reported, and there are already available templates developed for reporting WES findings.

Still, it might take a decade before the new technologies will enter mainstream medicine. The main reason for the slow adoption of genomic information, besides regulatory barriers, is the clinicians’ readiness and acceptance of incorporating the NGS findings into their routine case management (Metcalfe et al., 2009; Vassy et al., 2015a). Having clinician-friendly reporting will definitely speed-up the uptake process (Vassy et al., 2015b; Manolio, 2017).

In recent years, some companies have made inroads into NGS clinical reporting using omics analytics powered by AI technologies. In the industry sector of integrated WGS/WES clinical reporting, there are at least four commercial entities that offer clinician-friendly analytics and reporting:

• Qiagen (Ingenuity Variant Analysis and Ingenuity Pathway Analysis) (QIAGEN, 2018b)
• Golden Helix (VarSeq, VSCkinical) (Golden Helix, 2017)
• Advaita (iVariant/iPatway/iBio Guides) (ADVAITA, 2018)
• Lifemap Sciences (TGex^TM^, 2018)

All four solutions are available through a Web-based interface and offer clinical prioritization (using as input Variant Calling File – VCF) that deploys some aspect of AI. Qiagen applications (Annovar is part of the suite) are the clear leaders, as traditionally most genomic laboratory companies use their offerings (Krämer et al., 2014). In terms of innovation, the sheer depth of knowledge and the ease of generating clinical reports makes Lifemap Sciences and its clinical exome analysis suite (TGex) the top scorer (Ben-Ari Fuchs et al., 2016; Stelzer et al., 2016a,b). It is by far the most clinician-friendly WES analysis pipeline and reporting. It is also one of the most affordable on the market. It compiles over 110 different biological databases, ranging from gene ontology (GO) and biological pathways, through network interdependencies, transcriptional expression, and ending at phenotype essentialities. Because of its coverage of omics data (width and depth), many bioinformatics analytical tools utilize their resources including the companies mentioned above.

AI is most commonly deployed at two levels within clinical bioinformatics: *in silico* gene damage scoring (mostly Markov Hidden Model) (Liu et al., 2016b; McLaren et al., 2016; Feng, 2017) and prioritization and phenotype scoring, where various text mining algorithms are adopted. Other than that, AI is still a research tool until large longitudinal data, and more robust informatics frameworks are available. It is worth mentioning that one of the main strengths of AI in clinical practice is the area of image recognition (Alyass et al., 2015; He et al., 2017; Lytras and Papadopoulou, 2018). Many research studies are incorporating AI image processing with pathology and clinical imaging to improve diagnostic decision-making.

Artificial intelligence tools incorporating omics data are still a nascent development; they are a valuable addition to the existing bioinformatics application arsenal and a valuable connection between medical molecular geneticists and frontline clinicians.

## Future Considerations

**FIGURE 2:**
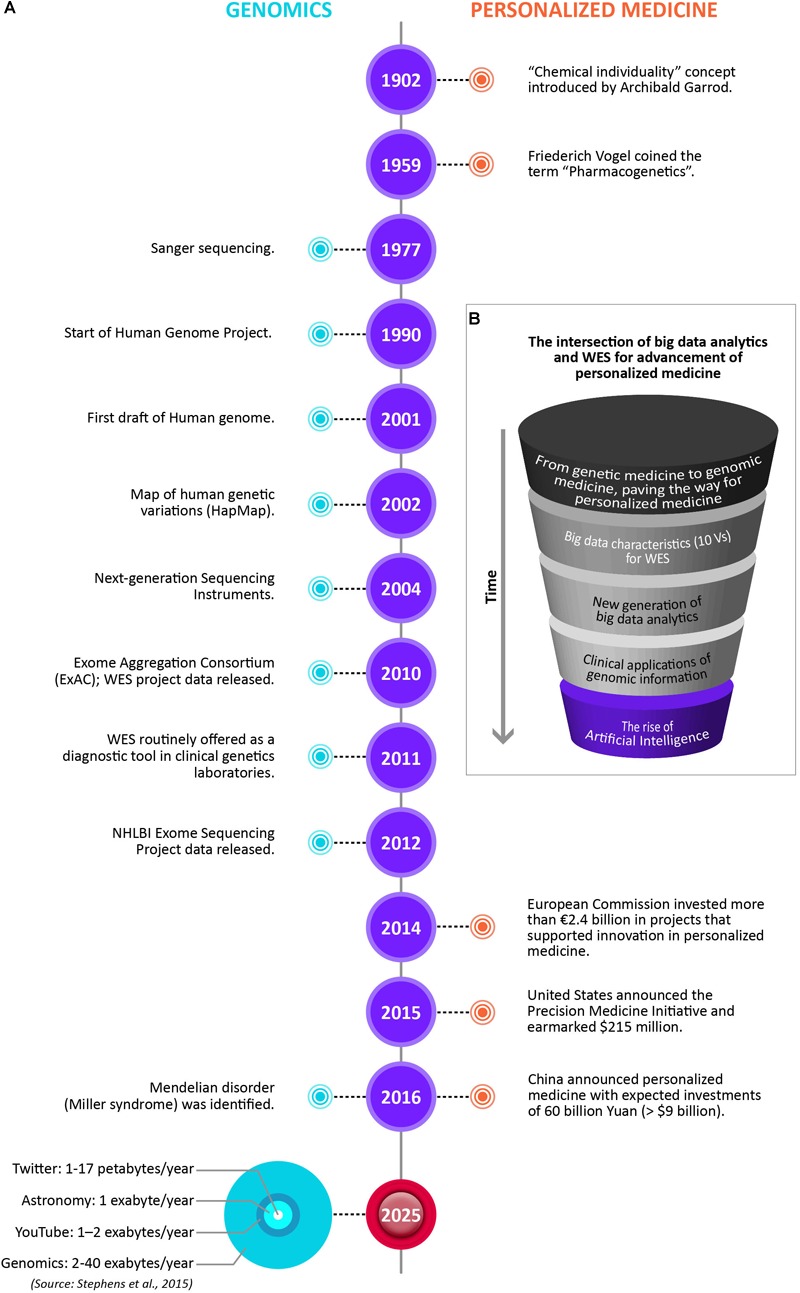
The changing paradigms of personalized medicine. **(A)** Notable timelines in Genomics and Personalized Medicine, including the data storage size for the four big data domains by 2025, with genomics either on par or the most demanding of the domains (Stephens et al., 2015). **(B)** The intersection of big data analytics and WES for advancement of personalized medicine. The drawings are not to scale.

The advancement of personalized medicine in many ways is being driven by the intersection of big data analytics and WES. Figure 2 illustrates the changing paradigms of personalized medicine. Notable timelines in Genomics and Personalized Medicine are showcased, including the data storage size of the four big data domains by 2025, with genomics either on par or the most demanding of the domains (Stephens et al., 2015). However, there are many barriers still for WES to have a wider use in mainstream medical practice. The major challenges include results reproducibility, reporting standards, and affordability.

### Results Reproducibility

A recent study conducted by the American College of Medical Genetics and Genomics (ACMG) showed significant variability in results reproducibility between different genetic laboratories. As a result, the Association together with industry players have developed the standards for genetic tests assessment. Although it is still voluntary, laboratories are encouraged to validate their products against industry standards (Amendola et al., 2016).

### Reporting Standards

Standards for reporting results of genetic tests have also been developed by ACMG; however, they only address pathogenic variants detected in ACMG recommended 59 genes. The Harvard School of Medicine, in collaboration with Healthcare Partners, designed a more comprehensive template for reporting results related to genetic diseases, polygenic/multifactorial diseases, and pharmacogenetics (Vassy et al., 2015b). It has to be noted that the polygenic risk score is based on odds ratios reported in the GWAS database (Table 1). Conditions with variants without odds ratios or *P*-value score cannot be assessed. The main objective of the reporting template was to present genomic tests results in a clinician-friendly manner so that it can be even used at all levels of health care services, including primary care physicians (Metcalfe et al., 2009; Harvard Medical School, 2015; Manolio, 2017).

For omics based genomic analysis, no standard template exists and each laboratory reports use their own standards. As the goal of multi-omics prioritization is to detect variants, functional effect on genes and possible genes’ phenotypic essentiality, the practical way of reporting would be to focus on the Loss of Function/Partial Loss of Function (LOF/PLOF) and phenotype essentiality scoring.

### Affordability

Generally, genetic tests are expensive (Topol, 2014; Kong et al., 2015; Bomba et al., 2017; He et al., 2017). The tests can be divided into two technological groups: genotyping and sequencing. Genotyping tests are less costly (USD 100–400), but analyze a limited number of variants, genes (regions). Since scientific progress produces new information on a daily basis, genotyping tests need to be repeated when current findings are included. Sequencing, on the other hand, is much more expensive (>USD 400) but detects variants at any location within the queried region. Also, the clinical utility of sequencing is higher, and there is no need for repeated tests. The cost of WGS is still prohibitive (>USD 1000) for routine application in medical practice. It also produces a large amount of unusable data. A more practical approach is the sequencing of all coding and flanking regions (WES), which covers between 3 and 6% of the genome, and the cost for commercial use can be as low as USD 400 (Macrogen Korea, 2017). The affordability, actionable data, no repeated tests required, and lower junk data makes WES a genetic test of choice (Vissers et al., 2017). Unfortunately, the total cost of WES clinical interpretation is still high (>USD 1000), which makes it more of a premium service rather than first line modality. The affordability of WGS and WES sequencing tests can be dramatically increased provided health insurance companies agree to reimburse the cost.

## Author Contributions

PS, CKO, MHTL, YMP, AMK, and HSO contributed in the writing of the manuscript.

## Conflict of Interest Statement

CKO is an employee of AstraZeneca UK Limited with an interest in the deployment of WES for personalized medicine. The remaining authors declare that the research was conducted in the absence of any commercial or financial relationships that could be construed as a potential conflict of interest.
